# Role of the Small GTPase Rho3 in Golgi/Endosome Trafficking through Functional Interaction with Adaptin in Fission Yeast

**DOI:** 10.1371/journal.pone.0016842

**Published:** 2011-02-03

**Authors:** Ayako Kita, Cuifang Li, Yang Yu, Nanae Umeda, Akira Doi, Mitsuko Yasuda, Shunji Ishiwata, Atsushi Taga, Yoshitaka Horiuchi, Reiko Sugiura

**Affiliations:** 1 Laboratory of Molecular Pharmacogenomics, School of Pharmaceutical Sciences, Kinki University, Higashi-Osaka, Japan; 2 Laboratory of Pharmaceutical Analytical Chemistry, School of Pharmaceutical Sciences, Kinki University, Higashi-Osaka, Japan; 3 Kinki University Life Science Research Institute, Osakasayama, Japan; University of Minnesota, United States of America

## Abstract

**Background:**

We had previously identified the mutant allele of *apm1^+^* that encodes a homolog of the mammalian µ1A subunit of the clathrin-associated adaptor protein-1 (AP-1) complex, and we demonstrated the role of Apm1 in Golgi/endosome trafficking, secretion, and vacuole fusion in fission yeast.

**Methodology/Principal Findings:**

In the present study, we isolated *rho3^+^*, which encodes a Rho-family small GTPase, an important regulator of exocystosis, as a multicopy-suppressor of the temperature-sensitive growth of the *apm1-1* mutant cells. Overexpression of Rho3 suppressed the Cl^−^ sensitivity and immunosuppressant sensitivity of the *apm1-1* mutant cells. Overexpression of Rho3 also suppressed the fragmentation of vacuoles, and the accumulation of v-SNARE Syb1 in Golgi/endosomes and partially suppressed the defective secretion associated with *apm1*-deletion cells. Notably, electron microscopic observation of the *rho3*-deletion cells revealed the accumulation of abnormal Golgi-like structures, vacuole fragmentation, and accumulation of secretory vesicles; these phenotypes were very similar to those of the *apm1*-deletion cells. Furthermore, the *rho3*-deletion cells and *apm1*-deletion cells showed very similar phenotypic characteristics, including the sensitivity to the immunosuppressant FK506, the cell wall-damaging agent micafungin, Cl^−^, and valproic acid. Green fluorescent protein (GFP)-Rho3 was localized at Golgi/endosomes as well as the plasma membrane and division site. Finally, Rho3 was shown to form a complex with Apm1 as well as with other subunits of the clathrin-associated AP-1 complex in a GTP- and effector domain-dependent manner.

**Conclusions/Significance:**

Taken together, our findings reveal a novel role of Rho3 in the regulation of Golgi/endosome trafficking and suggest that clathrin-associated adaptor protein-1 and Rho3 co-ordinate in intracellular transport in fission yeast. To the best of our knowledge, this study provides the first evidence of a direct link between the small GTPase Rho and the clathrin-associated adaptor protein-1 in membrane trafficking.

## Introduction

In eukaryotic cells, Rho family small GTPases play a crucial role in polarized growth through reorganization of the actin cytoskeleton and the regulation of secretory vesicle transport [1]. Although detailed knowledge is available on the role of Rho family proteins in the actin cytoskeleton, various functional aspects of the Rho signaling pathway with regard to membrane trafficking are relatively unknown.

Recently, Rho GTPase proteins have been attracting increasing attention for their role in exocytosis [2]. In mast cells, recombinant Rac and Rho proteins stimulate the exocytosis of secretory granules [1]. RhoD and RhoB are localized in endocytic vesicles, and RhoD regulates endosome dynamics through Diaphanous-related Formin and Src tyrosine kinase [3]. In the budding yeast *Saccharomyces cerevisiae*, Rho3 appears to influence cell growth by regulating polarized secretion as well as the actin cytoskeleton by interacting with Exo70 and Myo2 [4], [5]. Thus, the Exo70 subunit of the exocyst is an effector of Rho3 in polarized exocytosis [6]. In the fission yeast *Schizosaccharomyces pombe*, Rho3 is implicated in polarized cell growth through both Formin [7] and by modulating exocyst function [8]. However, little is known about the role of Rho3 in Golgi/endosome function in fission yeast.

We previously identified a mutant allele of the *apm1* ^+^ gene that encodes a µ1 subunit of the adaptor protein complex, and characterized the role of Apm1 in Golgi/endosome trafficking, vacuole fusion, and secretion in fission yeast [9]. In order to gain further insight into the function of Apm1, we screened for a multi-copy suppressor of *apm1-1* mutant cells and identified Rho3, a member of the Rho GTPase family.

In the present study, we show that in addition to its well-known role for the regulation of exocytosis, Rho3 plays an important role in Golgi/endosome trafficking. Notably, Rho3 forms a complex with Apm1 and with other subunits of the clathrin-associated adaptor protein-1 complex and suppresses the deletion cells of all the subunits of the AP-1 complex. To the best of our knowledge, the present study provides the first evidence of a direct link between the small GTPase Rho3 and the clathrin-associated adaptor protein-1 in membrane trafficking.

## Materials and Methods

### Strains, Media, Genetic, and Molecular Biology Methods

*S. pombe* strains used in this study are listed in Table 1. The complete and minimal media were yeast extract-peptone-dextrose (YPD) and Edinburgh minimal medium (EMM), respectively [10]. Standard genetic and recombinant DNA methods [11] were used except where stated otherwise. FK506 was provided by Astellas Pharma Inc. (Tokyo, Japan). Genome DNA clones were provided by the National Bio Resource Project, Yeast Genetic Resource Center (Graduate School of Science, Osaka City University).

**Table 1:** *Schizosaccharomyces pombe* strains used in this study.

Strain	Genotype	Reference
HM123	*h^−^ leu1-32*	Our stock
KP1248	*h^−^ leu1-32 ura4-294*	Our stock
KP356	*h^−^ leu1-32 cis1-1/apm1-1*	Kita *et al.*, 2004
KP630	*h^−^ leu1-32 ura4-D18 apm1*::*ura4^+^*	Kita *et al.*, 2004
KP1375	*h^−^ leu1-32 ura4-D18 rho3*:: *ura4^+^*	This study
KP2035	*h^−^ leu1-32 ura4-D18 nmt1* GFP*-syb1^+^*::*ura4^+^*	Kita *et al.*, 2004
SP962	*h^−^ leu1-32 ura4-D18 nmt1* GFP*-rho3^+^*::*ura4^+^*	This study
SP695	*h^−^ leu1-32 ura4-D18 nmt1* GFP*-syb1^+^*::*ura4^+^ apm1*::*ura4^+^*	This study
SP980	*h^−^ leu1-32 ura4-D18 apm1-*GFP::*leu1^+^*	This study
KP427	*h^−^ leu1-32 ura4-D18 apl2*::*ura4^+^*	Ma *et al.*, 2009
KP3463	*h^−^ leu1-32 ura4-D18 apl4*::*ura4^+^*	Ma *et al.*, 2009
KP3391	*h^−^ leu1-32 ura4-D18 aps1*::*ura4^+^*	Ma *et al.*, 2009

### Cloning and Tagging of the *rho3* ^+^ and *apm1* ^+^ genes

The *apm1-1* mutant was transformed using an S. *pombe* genomic DNA library constructed in the vector pDB248 [12]. Leu+ transformants were replica-plated onto YPD plates at 36°C, and the plasmid DNA was recovered from transformants that exhibited plasmid-dependent rescue. The plasmids that complemented the temperature sensitivity of the *apm1-1* mutant were cloned and sequenced. The suppressing plasmids fell into 2 classes; one containing *apm1^+^*, and the other containing *rho3^+^* (SPAC23C4.08). The *rho3^+^* gene was amplified by polymerase chain reaction (PCR) using the genomic DNA of wild-type cells as a template. The sense and antisense primers were 5′-CGC GGA TCC CAT ATG TCA AGC TGT TTC GG-3′, and 5′-CG GGA TCC TCA AGC AAT GAT ACA TCC GGT A-3′, respectively. The amplified product containing *rho3^+^* was digested with *Bam*HI, and the resulting fragment was subcloned into BlueScriptSK (+) (Stratagene). For ectopic expression of proteins, we used the thiamine- repressible *nmt1* promoter [13]. Expression was repressed by the addition of 4 µM thiamine to EMM. In order to express green fluorescent protein (GFP)-Rho3, the complete open reading frame (ORF) of *rho3^+^* was ligated to the N-terminus of the GFP carrying the S65T mutation [14]. In order to obtain the chromosome-borne *nmt1* GFP-Rho3, the fused gene was subcloned into the vector containing the *ura4^+^* gene locus of KP1248 as described. The Apm1-mCherry fusion construct was confirmed by restriction digestion and sequence analysis. The functionality of the obtained protein was verified by complementation of the Δ*apm1* cells and by localization of Apm1-mCherry.

### Gene Disruption of *rho3^+^*

A one-step gene disruption by homologous recombination was performed as described before [15]. The *rho3*::*ura4^+^* disruption was constructed as follows. The *Bam*HI fragment containing *rho3^+^* was subcloned into the *Bam*HI site of pUC119, yielding pUC-rho3. The pUC-rho3 plasmid was cleaved at the single *Eco*RV site located in the *rho3^+^* coding region and was then ligated with the *Bgl*II linker, which yielded pUC-rho3*Bgl*II. The resulting DNA was then ligated to the 1.8-kb *Bam*HI fragment of the *S. pombe ura4^+^* DNA to create pUC-*rho3*::*ura4^+^*. The *Bam*HI fragment containing the disrupted *rho3^+^* gene was transformed into haploid cells. Stable integrants were selected on medium lacking uracil, and disruption of the gene was examined by genomic Southern hybridization (data not shown).

### Site-directed Mutagenesis

The site-directed mutagenesis of Rho3 was performed using the Quick Change Site-Directed Mutagenesis Kit (Stratagene). In the PCR reaction, the following mutant primers were used to change specific amino acids: 5′-CTA AGC ATT CTA ACC GTA AAT TAT AGT GGC CGC TGG TAA AAC CAG TTT G-3′ and 5′-CAA ACT GGT TTT ACC AGC GGC CAC TAT AAT TTA CGG TTA GAA TGC TTA G-3′ were used to change Gly22 to Val; 5′-GTA AAT TAT AGG TGC TGC TGG TAA AAA CAG TTT GTT AAA TGT ATT TAC TAA G-3′ and 5′-CTT AGT AAA TAC ATT TAA CAA ACT GTT TTT ACC AGC AGC ACC TAT AAT TTA C-3′ were used to change Thr27 to Asn; 5′-CCG TTA TCT AAC ACC TAA AAA TAG ATT TGT TAA CTA CAT TCA TGA TAT C TT TGT CG-3′ and 5′-CGA CAA AGA TAT CAT GAA TGT AGT TAA CAA ATC TAT TTT TAG GTG TTA GAT AAC GG-3′ were used to change Glu48 to Val.

### Microscopy and Miscellaneous Methods

Light microscopy methods (e.g., fluorescence microscopy) were performed as described [9]. FM4-64 labeling, localization of GFP-Syb1, measurement of the acid phosphatase secretion, and conventional electron microscopy were performed as described previously [9].

### Affinity Capillary Electrophoresis for In Vitro Protein-Protein Binding Assay

Affinity capillary electrophoresis (ACE) was performed as reported in our previous studies [16], [17]. Capillary electrophoresis was performed by assembling a HER-30PI high-voltage power supply from Matsusada Precision Devices (Kusatsu, Shiga, Japan) and a laser-induced fluorescence (LIF) detector from GL Sciences Inc. (Shinjuku-ku, Tokyo, Japan). The FunCap-CE/Type C (total length, 80 cm; effective length, 40 cm; internal diameter, 50 µm) from GL Sciences Inc. for use as a carboxylated capillary was used as a separation column. A stock solution of phosphate buffer was prepared by adding 50 mM disodium hydrogen phosphate aqueous solution to 50 mM sodium dihydrogen phosphate solution until a pH of 6.8 was achieved on a pH meter. Background electrolytes (BGEs) containing proteins were prepared by dissolving GST-fused Apm1 or GST-fused Rho3 (as a negative control) at a concentration of 200 µg/ml or 100 µg/ml. Here, we used GST-Rho3 instead of GST as a control, because the differences in the molecular masses of Apm1-GST and GST could affect the accuracy of the assay. For ACE, the proteins in the BGEs were diluted to appropriate concentrations by using the same buffer. GFP-fused proteins (wild-type Rho3 and its mutant versions) in the BGEs containing GST-fused proteins were analyzed at various concentrations. Degassed BGEs were filtered through a 0.45-µm membrane filter before use. Sample solutions were introduced from the anodic end of the capillary for 10 s by using hydrostatic method. A potential of 15 kV was applied between both ends of the capillary. The capillary tube was conditioned by rinsing with an aqueous solution of 1 M sodium chloride for 2 min followed by rinsing with an electrophoretic solution for 5 min before introducing each sample. The proteins were detected by monitoring the total fluorescence intensity at wavelength 500–600 nm induced by laser at 473 nm. Capillary electrophoresis analyses were performed at 25±0.1°C. In this method, the migration times were reflective of the molar ratios of the complex types. Therefore, migration time-shifts of GFP-fused Rho3 proteins indicate the strength of the interaction between the protein sample and GST-proteins as ligands in BGEs.

## Results

### Identification of *rho3* ^+^ as a multicopy suppressor of *apm1-1*

**Figure 1:**
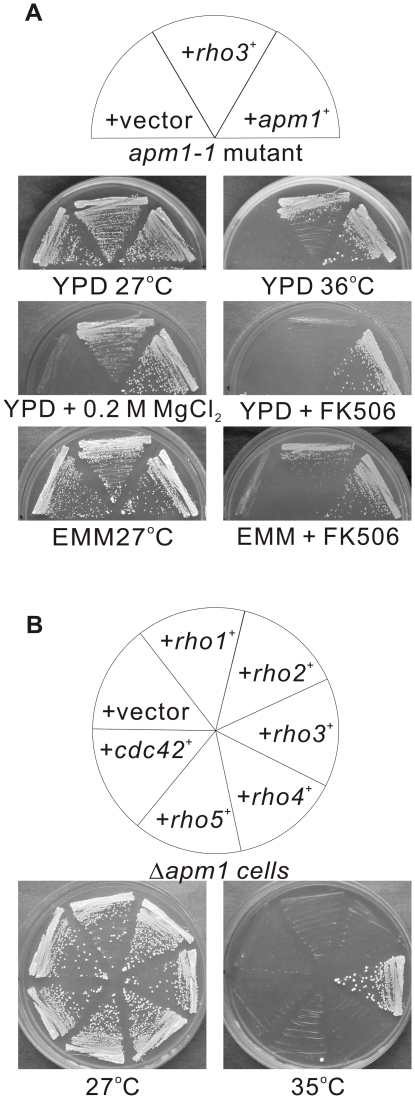
Isolation of Rho3 as a multicopy suppressor of *apm1* mutant cells. (A) The *apm1* mutant cells (*apm1-1*) were transformed with either the pDB248 multicopy vector, the vector containing *apm1^+^* or the vector containing *rho3^+^*. Cells were then streaked onto plates containing YPD, YPD plus 0.2 M MgCl_2_, YPD plus 0.5 µg/mL FK506, EMM, or EMM plus 0.5 µg/mL FK506 and then incubated for 4 d at 27°C or for 3 d at 36°C, respectively. (B) Cells transformed with the multicopy vector pDB248, or the genome DNA clones containing *rho1^+^*, *rho2^+^*, *rho3^+^*, *rho4^+^*, *rho5^+^*, or *cdc42^+^* were streaked onto plates containing YPD and incubated for 4 d at 27°C or 3 d at 35°C, respectively.

The *apm1-1* mutants grew at the permissive temperature of 27°C, but failed to grow at the restrictive temperature of 36°C, in the media containing either 0.2 M MgCl_2_ or FK506, a specific inhibitor of calcineurin phosphatase (Figure 1A, +vector) [9]. In order to identify novel genes that are involved in Apm1 function, we screened a fission yeast genomic library to isolate genes that when overexpressed, could suppress the temperature sensitivity of *apm1-1* mutant cells. One of the genes identified was *rho3^+^* which encodes a member of the Rho family of small GTPases. Overexpression of *rho3^+^* suppressed both the temperature (Figure 1A; +*rho3* ^+^, 36°C), and Cl^−^ sensitivity (Figure 1A, +*rho3* ^+^, +0.2 M MgCl_2_) of the *apm1-1* mutants. Overexpression of the *rho3^+^* gene only weakly suppressed the FK506 sensitivity of *apm1-1* mutants in the YPD medium (Figure 1A; +*rho3* ^+^, YPD + FK506). However, *rho3^+^* overexpression more strongly suppressed the FK506 sensitivity of *apm1-1* mutants in the EMM medium (Figure 1A, +*rho3* ^+^, EMM + FK506).

In order to determine the specificity of Rho3 in the suppression of *apm1* mutants, we investigated the effect of other genes that encode members of the Rho family of small GTPases present in the fission yeast genome. In order to test the suppression capabilities of all 6 of the fission yeast Rho family members, Δ*apm1* cells transformed with *rho1^+^*, *rho2^+^*, *rho3^+^*, *rho4^+^*, *rho5^+^*, or *cdc42^+^* were tested for growth at 35°C. The results clearly indicated that this property is highly specific to Rho3, because the overexpression of Rho3, but none of the other Rho family members, could suppress the temperature sensitivity of Δ*apm1* cells (Figure 1B).

### Rho3 suppresses defective membrane trafficking in Δ*apm1* cells

**Figure 2:**
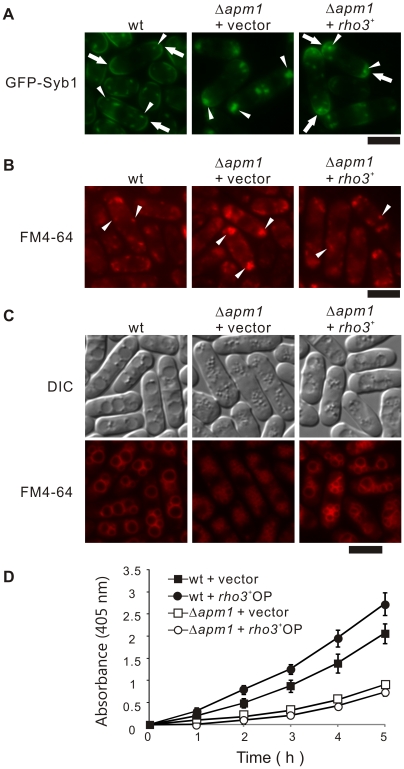
Rho3 suppressed various phenotypes associated with Apm1-deletion cells. (A) Rho3 suppressed the defective localization of GFP-Syb1 in Δ*apm1* cells. Wild-type cells (wt) and Apm1-deletion (Δ*apm1*) expressing chromosome-bone GFP-Syb1 cells transformed with pDB248 or the vector containing *rho3^+^* was cultured in YPD medium at 27°C. The GFP-Syb1 localization was examined under the fluorescence microscope. Bar 10 µm. (B) Rho3 suppressed the defective localization of FM4-64 in Δ*apm1* cells. Wild-type (wt) and Apm1-deletion cells (Δ*apm1*) transformed with pDB248 or the vector containing *rho3^+^* were cultured in YPD medium at 27°C. Cells were collected, labeled with FM4-64 fluorescent dye for 5 min, resuspended in water, and examined by fluorescence microscopy. Bar 10 µm. (C) Wild-type (wt) and Apm1-deletion cells (Δ*apm1*) transformed with pDB248 or the vector containing *rho3^+^* cultured in YPD medium at 27°C. Cells were collected, labeled with FM4-64 fluorescent dye for 60 min, resuspended in water, and examined by fluorescence microscopy. Bar 10 µm. (D) Wild-type cells and Δ*apm1* cells, which were transformed with either the pDB248 vector or the *rho3^+^*-containing vector, were assayed for acid phosphatase activity as indicated in the Materials and Methods section. Values from 3 independent experiments were plotted as means ± S.D.

In order to investigate if Rho3 overexpression could rescue the membrane-trafficking defects in Δ*apm1* cells, we examined the effect of Rho3 overexpression on the abnormal localization of GFP-fused Syb1 in Δ*apm1* cells. Syb1, the synaptobrevin in fission yeast, is a vesicle-associated membrane protein that can be copurified with secretory vesicles [18]. As a secretory vesicle SNARE, Syb1 is expected to cycle between the cell surface and the endocytic pathway. In order to assess the Golgi-to-endosome or Golgi-to-plasma membrane trafficking pathway, the localization of GFP-Syb1 was monitored. The fluorescence of GFP-Syb1 in wild-type cells could be enriched in the medial region or cell ends, and was detected in Golgi/endosomes (Figure 2A, wt, arrows). In contrast, GFP-Syb1 failed to localize on the cell surface, medial regions, or the Golgi/endosomes in Δ*apm1*cells; instead, they were observed as large brightly fluorescent dots in the cytoplasm at 27°C (Figure 2A, Δ*apm1* + vector, arrowheads) [9]. Notably, GFP-Syb1 was visible at the cell ends in Δ*apm1* cells harbouring *rho3* ^+^ (Figure 2A, Δ*apm1* + *rho3* ^+^, arrows), and the accumulation of large dots in the cytosol was greatly improved (Figure 2A, Δ*apm1*+*rho3* ^+^, arrowheads). Also, FM4-64 dots at an early stage of endocytosis was observed as large clusters in Δ*apm1* cells, whereas Rho3 overexpression recovered the normal dot like structures almost similar to those observed in wild-type cells (Figure 2B, arrowheads).

Next, we examined the effect of Rho3 overexpression on vacuole fusion, because fragmented vacuoles were observed in the Δ*apm1* cells [9]. In order to do so, we utilized FM4-64 a vital fluorescent dye that is internalized in living cells and then accumulates in the vacuoles. After the cells were labeled with FM4-64 for 60 min, collected, washed, and resuspended in water for 90 min, the wild-type cells had large prominent vacuoles resulting from vacuole fusion [19] (Figure 2C, wt). On the other hand, vacuoles remained small and numerous in Δ*apm1* cells (Figure 2C, Δ*apm1* + vector), indicating a defect in vacuole fusion. In contrast, Δ*apm1* cells transformed with *rho3^+^* contained large vacuoles, indicating that Rho3 overexpression partially but clearly suppressed the defects in vacuole fusion observed in Δ*apm1* cells (Figure 2C, Δ*apm1* + *rho3^+^*).

Next, we examined the effect of *rho3^+^* overexpression on the defects in secretion associated with Δ*apm1* cells [9]. Overexpression of *rho3* ^+^ in wild-type cells stimulated greater secretion of acid phosphatase than the control vector (Figure 2D, wt + *rho3* ^+^ OP). Notably, overexpression of *rho3* ^+^ partially but significantly stimulated secretion in Δ*apm1* cells (Figure 2D, Δ*apm1* + *rho3* ^+^ OP, Table 2).

**Table 2:** Analysis of acid phosphatase secretion and effect of Rho3 overproduction.

	wt+vector	wt+*rho3^+^*OP	Δ*apm1*+vector	Δ*apm1*+*rho3^+^*OP
0 h	0±0	0±0	0±0	0±0
1 h	0.195±0.082	0.306±0.097	0.009±0.081	0.103±0.032
2 h	0.487±0.129	0.788±0.123	0.104±0.126	0.179±0.024
3 h	0.870±0.172	1.250±0.129	0.208±0.081	0.318±0.028
4 h	1.384±0.262	1.949±0.229	0.424±0.076	0.556±0.014
5 h	2.057±0.278	2.726±0.311	0.730±0.097	0.898±0.014

Wild-type cells and Δ*apm1* cells, which were transformed with either the pDB248 vector or the *rho3^+^*-containing vector, were assayed for acid phosphatase activity as indicated in the Materials and Methods section. Values from 3 independent experiments are expressed as means ± standard deviation.

### Electron microscopic analysis of Δ*rho3* cells

**Figure 3:**
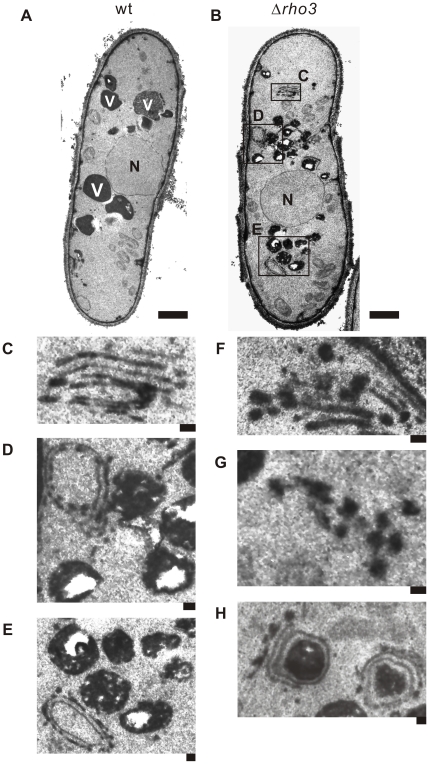
Electron microscopic analysis of Δ*rho3* cells. Rho3- deletion cells (Δ*rho3*) exhibit large Golgi structures and accumulate membranous structures. Wild-type (wt) (A) and Rho3-deletion cells (Δ*rho3*) (B) were analyzed by electron microscopy at 27°C. The boxed regions (C, D, and E) in (B) are enlarged. N, nucleus; V, vacuoles. Bar: 0.1 µm. (C) Multi-lamellar Golgi structures. (D) An abnormal membranous structure of a Berkeley body. Bar: 0.1 µm. (E) Fragmented vacuoles in Δ*rho3* cells. Bar 0.1 µm. (F), (G) Putative post-Golgi vesicles around Golgi structures. Bar 0.1 µm. (H) Abnormal electron-dense membranous structures closely connected to the Golgi structure. Bar: 0.1 µm.

As mentioned above, Rho3 overexpression suppressed defects in membrane trafficking of Δ*apm1* cells especially phenotypes associated with Golgi/endosomes, and vacuoles; this suggests that Rho3 may be related to these functions in addition to its well-known role in secretion. This prompted us to analyze Δ*rho3* cells with electron microscopy. In general, electron microscopic analysis of mutants that exhibit defects in membrane trafficking reveals the accumulation of organelles or vesicular intermediates of the compartments that precede the step in which they first function [20]–[22]. Wild-type and Δ*rho3* cells were cultured at 27°C and examined by electron microscopy in order to determine whether Δ*rho3* cells accumulate such structures (Figure 3A and B). Notably, Golgi structures in Δ*rho3* cells were thick, swollen, and were frequently multi-lamellar (Figure 3C and F). Moreover, large vesicular structures (>100 nm in diameter) associated with Golgi stacks were observed in Δ*rho3* cells (Figure 3F), suggesting that Rho3 is involved in vesicle formation at the trans-Golgi network. The accumulation of abnormal electron-dense membranous structures (also known as Berkeley bodies) was also observed in Δ*rho3* cells (Figure 3–D, E, and H). Abnormal membranous structures related to Golgi structures were observed in Δ*rho3* cells (∼5%) but were seldom observed in wild-type cells (Figure 3A). Δ*rho3* cells also accumulated putative large post-Golgi vesicles ranging from 100 to 150 nm in size that were intensely stained after permanganate fixation (Figure, 3G); on the other hand, accumulation was almost negligible in wild-type cells (Figure 3A). Another striking feature of Δ*rho3* cells is that their vacuoles were fragmented (Figure 3-B, D, and E) compared to wild-type cells (Figure 3A).

It should be noted that the abnormal findings seen in the electron micrographs of Δ*rho3* cells are similar to previously reported findings in *ypt3-i5* mutant [22] and Δ*apm1* cells [9]. These suggest the novel role of Rho3 in that it is functionally associated with the Golgi and vacuoles, in addition to its role in the secretory pathway.

### Δ*rho3* cells exhibit phenotypes similar to those of Δ*apm1* cells

**Figure 4:**
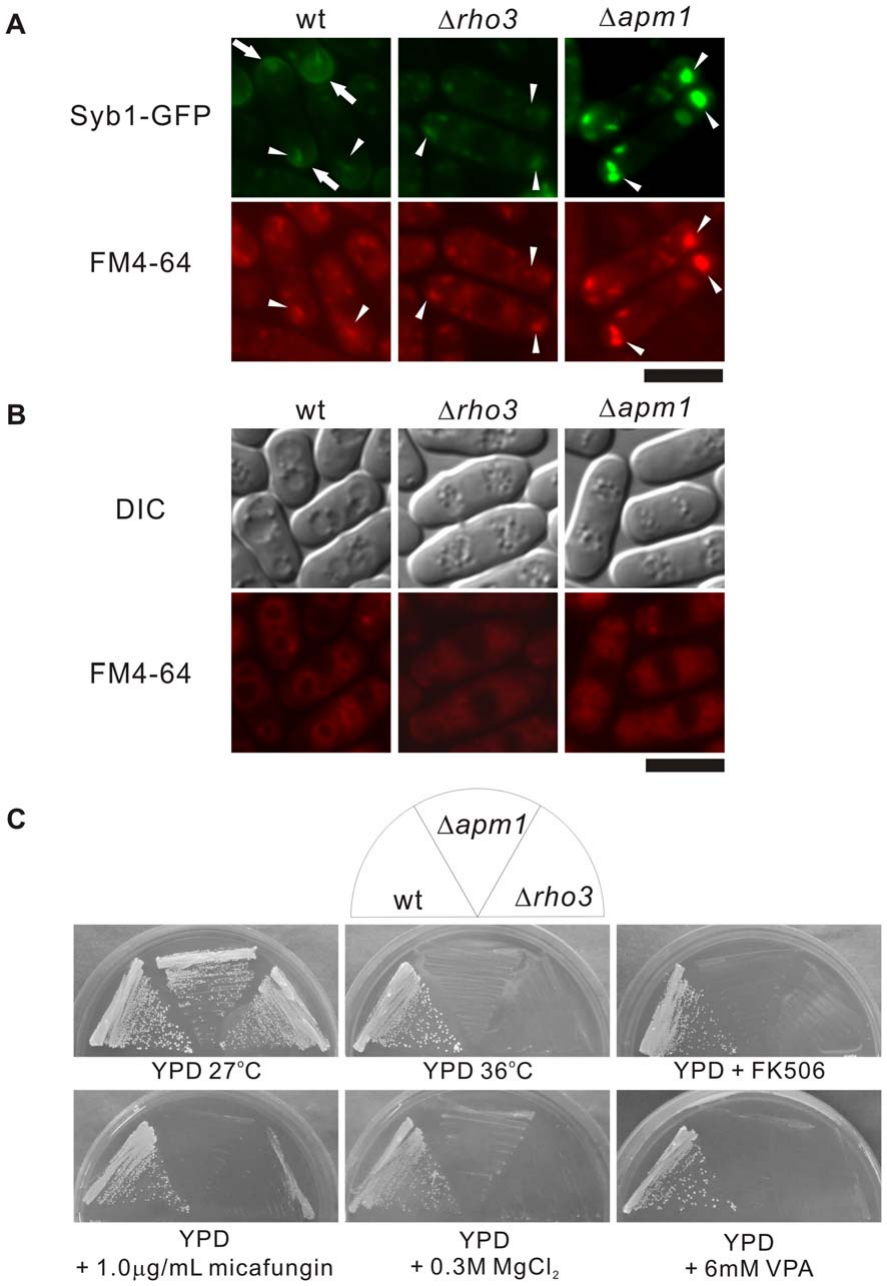
Rho3-deletion cells displayed phenotypes similar to those seen in Apm1-deletion cells. (A) Wild-type (wt), Rho3-deletion (Δ*rho3*), and Apm1-deletion cells (Δ*apm1*) expressing chromosome-bone GFP-Syb1 cultured in YPD medium at 27°C were incubated with FM4-64 fluorescent dye for 5 min at 27°C to visualize Golgi/endosomes. GFP-Syb1 localization and FM4-64 fluorescence were examined under a fluorescence microscope. Bar 10 µm. (B) Wild-type (wt), Rho3-deletion (Δ*rho3*), and Apm1-deletion cells (Δ*apm1*) were cultured in YPD medium at 27°C. Cells were collected, labeled with FM4-64 fluorescent dye, resuspended in water, and examined by fluorescence microscopy. Photographs were taken after 60 min. Bar: 10 µm. (C) Wild-type (wt), Rho3-deletion (Δ*rho3*), and Apm1-deletion cells (Δ*apm1*) were streaked onto plates containing YPD or YPD plus 1.0 µg/mL micafungin, 0.5 µg/mL FK506, 0.3 M MgCl_2_, and 6 mM valproic acid, and were then incubated at 27°C for 4 d or at 36°C for 3 d.

The above results strongly suggest that defects in membrane trafficking in Δ*apm1* and Δ*rho3* cells are closely related; this prompted us to examine the phenotypes of Δ*rho3* cells in greater detail. First, we visualized Syb1 as a GFP-fusion protein in Δ*rho3* cells. As shown above, in Figure 4A, the fluorescence of GFP-Syb1 in wild-type cells was detected as punctuate structures in the cytoplasm that co-localize with FM4-64 dots at an early stage of endocytosis (arrowheads); this was also enriched in cell ends (arrows). In contrast, GFP-Syb1 failed to localize at the cell surface and the cell ends of Δ*rho3* and Δ*apm1* cells (Figure 4A), suggesting that Rho3 is also involved in the Golgi/endosome membrane trafficking pathway. It should be noted however, that Syb1 accumulation appears as large dots in the Δ*apm1* cells, but not in the Δ*rho3* cells, which suggests some differences in the phenotypes of these mutant cells. Moreover, Δ*rho3* cells exhibited defects in vacuolar fusion induced by osmotic stress in a similar fashion to Δ*apm1* cells. Hypotonic stress causes a dramatic fusion of vacuoles in wild-type, but not Δ*rho3* or Δ*apm1* cells (Figure 4B), indicating that Rho3 is required for vacuole fusion.

Furthermore, Δ*rho3* cells also exhibited cell wall-integrity defects as judged by the sensitivity to the cell-wall damaging agent, micafungin an inhibitor of (1,3)-β-D-glucan synthase. As shown in Figure 4C, Δ*rho3* and Δ*apm1* cells failed to grow on YPD plates containing 1.0 µg/mL micafungin, whereas wild-type cells were able to grow on these plates. We also examined the sensitivity of Δ*rho3* cells to high temperature (36°C), immunosuppressive drug FK506, MgCl_2_, and valproic acid (VPA) since Δ*apm1* cells failed to grow in the presence of these reagents or at a high temperature [23], [26]. As shown in Figure 4C, both Δ*rho3* and Δ*apm1* cells failed to grow on YPD plates containing 0.3 M MgCl_2_, FK506, and 6 mM valproic acid (VPA) or YPD plate incubated at 36°C. Thus, Δ*apm1* and Δ*rho3* cells have very similar phenotypes in terms of membrane-trafficking defects and sensitivity to ion- and cell wall-damaging agents.

### Functional relationship between Apm1 and Rho3 signaling

In order to investigate the functional relationship between Apm1 and Rho3 signaling, we constructed various mutant forms of Rho3 and examined their effects on the Δ*apm1* cells. The following mutants were included: a GDP-locked variant of Rho3, in which the conserved (among Rho3 proteins) Thr27 was replaced with Asn (Rho3T27N), a GTP-locked variant of Rho3 (Rho3G22V) in which the conserved Gly22 was replaced with Val, and an effector domain mutant Rho3 (Rho3E48V) in which the conserved Glu48 was replaced with Val.

**Figure 5:**
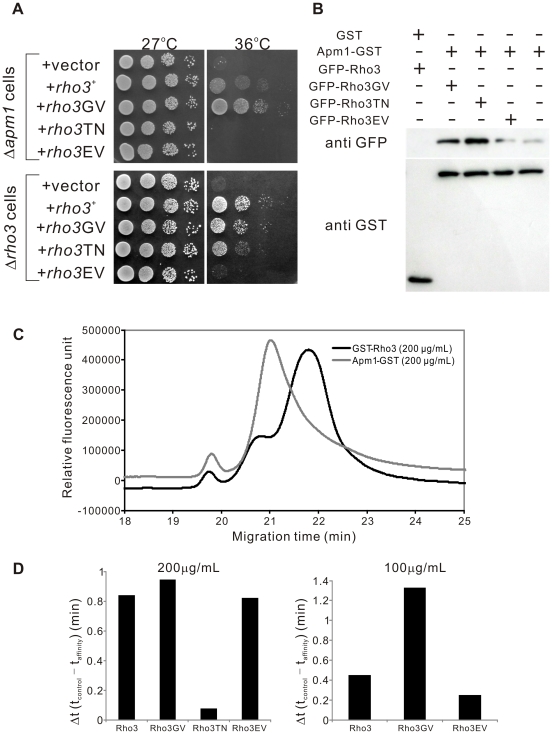
Functional and physical interaction between Rho3 and Apm1. (A) Rho3 suppressed Apm1-deletion cells (Δ*apm1*) in a GTP- and effector domain-dependent manner. Apm1-deletion cells (Δ*apm1*) were transformed with the pDB248 multi-copy vector or the vector containing *rho3^+^*, *rho3*GV, *rho3*TN, *and rho3*EV expressed from its endogenous promoter. Cells were spotted onto each YPD plate and then incubated for 3 d. Cells were spotted in serial 10-fold dilutions starting with OD_660_ = 0.6 of log-phase cells (5 µL). (B) Rho3 binds to Apm1 in a GTP- and effector domain-dependent manner. GST pull-downs performed with GST alone or Apm1-GST; cells expressing GFP-Rho3, GFP-Rho3GV, GFP-Rho3TN, or GFP-Rho3EV were collected, and the lysates were incubated with purified full-length Apm1 fused GST or the control GST protein. Proteins bound to glutathione beads were analyzed by SDS-PAGE and visualized by autoradiography. (C) Influence of Apm1 on the electropherograms of GFP-Rho3 protein determined by affinity capillary electrophoresis. Capillary, carboxylated fused silica (80 cm, 40 cm, 50 µm i. d.); BGE, 50 mM phosphate buffer (pH 6.8) containing Apm1-GST (gray line) or GST-Rho3 (black line: control) at a concentration of 200 µg/mL; applied voltage, 15 kV; temperature, 25°C; detection, laser-induced fluorescence detector (Ex, 473 nm; Em, 500-600 nm); sample introduction, hydrostatic method (10 cm ×10 s). (D) Comparison of the migration time-shifts in various Rho3 proteins. Δt (migration time-shift) is equal to t_control_ - t_affinity_, where t_control_ and t_affinity_ are the migration times of GFP-Rho3 or its mutant versions in the absence (control: GST-Rho3) or presence (affinity: Apm1-GST) of Apm1-GST, respectively. Left panel: The experiments were performed at a protein concentration of 200 µg/mL. Right panel: The experiments were performed at a protein concentration of 100 µg/mL.

The expression of the dominant-active Rho3G22V mutant suppressed the temperature-sensitive growth of Δ*apm1* cells more strongly than wild-type Rho3, suggesting that the GTP-bound form of Rho3 is the active form in the suppression of the Apm1 pathway (Figure 5A; Δ*apm1* cells, +*rho3* ^+^, +*rho3*GV). In contrast, the dominant-negative Rho3T27N an allele that is predicted to be in a GDP-bound form failed to suppress the temperature sensitivity of Δ*apm1* cells (Figure 5A; Δ*apm1* cells, +*rho3*TN). We also examined the effect of Rho3E48V an effector domain mutant of Rho3 on Δ*apm1* cells, using an analogue of the budding yeast *rho3-V51* mutant [4]. In particular, overexpression of Rho3E48V failed to suppress the temperature sensitivity of Δ*apm1* cells (Figure 5A; Δ*apm1* cells, +*rho3*EV). We also examined the effects of these plasmids on the temperature-sensitive growth of Δ*rho3* cells. Rho3 and Rho3G22V rescued the temperature-sensitive growth of Δ*rho3* cells, whereas Rho3T27N only weakly suppressed the growth defect (Figure 5A, Δ*rho3* cells). The growth rates between the Δ*rho3* cells harboring Rho3E48V and those bearing the vector were almost indiscernible (Figure 5A, Δ*rho3* cells).

Next, we examined whether various forms of Rho3 associate with Apm1. To do so, wild-type Rho3, the nucleotide-locked forms of Rho3 (GTPases in either the GTP-bound (Rho3G22V) or the GDP-bound conformation (Rho3T27N)), and the Rho3E48V effector domain mutant were fused to GFP and overexpressed using an inducible *nmt1* promoter; the induced cells were used to make lysates. These lysates were then used in binding experiments where purified full-length Apm1 was fused to glutathione-S-transferase (GST) or the control GST protein. The results showed that the Rho3G22V bound to Apm1 to a slightly greater degree than wild-type Rho3 (Figure 5B). In contrast, the binding of Apm1 with Rho3T27N or Rho3E48V was much weaker than that with wild-type Rho3 or Rho3G22V (Figure 5B). Therefore, the binding of Apm1 with Rho3 from yeast lysates demonstrates nucleotide-dependence and effector domain sensitivity. Thus, Rho3 functionally and physically associates with Apm1 in both a GTP- and an effector domain-dependent manner.

In a more rigorous investigation of the *in vitro* protein-protein interaction between Apm1 and Rho3, we performed an assay using affinity capillary electrophoresis [16], [17] (Materials and Methods). This method has been recently used in binding studies as the simplest method for reproducible and reliable analysis of the strength of the interactions between protein samples and ligands. In our study, the migration time-shifts of GFP-fused Rho3 or its mutant proteins indicated the strength of the interactions between GFP-Rho3 proteins and the purified Apm1-GST protein, which served as a ligand in the background electrolytes of capillary electrophoresis. We then examined the effects of ligand addition on the migration of the GFP-fused Rho3 protein. Figure 5C shows the electropherograms of GFP-Rho3 in the presence of GST-Rho3 or Apm1-GST proteins as ligands. As shown in Figure 5C, a migration time-shift (0.84 min) of the GFP-Rho3 peak was observed in the presence of Apm1-GST (200 µg/ml) in comparison with the findings for the control protein (GST-Rho3, 200 µg/ml), indicating that the Apm1-GST protein has affinity for the GFP-Rho3 protein.

Next, we performed *in vitro* binding experiments by utilizing various mutant versions of Rho3 and comparing the findings with those obtained for wild-type Rho3. As expected, the migration time-shift of Rho3GV was significantly greater (0.94 min) than that of wild-type Rho3 (Rho3) (0.84 min), and the migration time-shift of Rho3TN (0.08 min) was greatly reduced (Figure 5D). The migration time-shift of Rho3EV (0.82 min) was slightly lesser than that of wild-type Rho3 (Figure 5D: left panel). To allow more precise determination of the differences in the affinities of Rho3GV, Rho3EV, and wild-type Rho3, we performed binding experiments with a lower concentration of ligands (100 µg/ml). As shown in Figure 5D (right panel), the migration time-shift obtained with Rho3GV (1.33 min) was about 3 times greater than that obtained with wild-type Rho3 (0.45 min), and the migration time-shift obtained with Rho3EV (0.25 min) was about half of that obtained with wild-type Rho3. Thus, Rho3 was shown to form a complex with Apm1 in a GTP- and effector domain-dependent manner.

#### Localization of GFP-Rho3 at the Golgi/Endosomes

**Figure 6:**
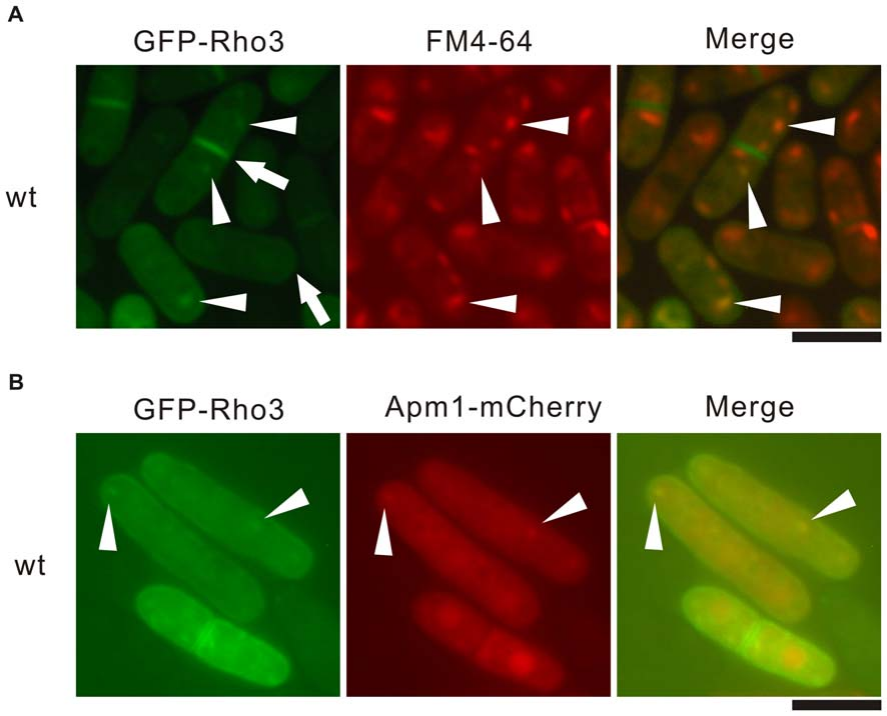
GFP-Rho3 localizes at the Golgi/endosome. (A) The colocalization of GFP-Rho3 with FM4-64 in wild-type cells. Wild-type (wt) cells, expressing chromosome-bone GFP-Rho3 under the control of the *nmt1* promoter, were examined by fluorescence microscopy under the repressed conditions. The cells were incubated with FM4-64 fluorescent dye for 5 min at 27°C to visualize Golgi/endosomes. The fluorescence of the adaptin subunit and FM4-64 was examined under the fluorescence microscope. Arrows indicate the plasma membrane and medial region. Arrowheads indicate the dot-like structures and Golgi/endosomes. Bar 10 µm. (B) The partial colocalization of GFP-Rho3 with Apm1-mCherry in wild-type cells. Wild-type cells expressing chromosome-borne GFP-Rho3 were transformed with pREP1-Apm1-mCherry. The cells were cultured and observed as described in Figure 6 (A). Bar: 10 µm.

Next, we re-examined the localization of Rho3. Previously, 2 studies have reported the localization of the Rho3 protein. Nakano *et al*. used antibodies raised against the budding yeast Rho3 protein and reported that Rho3 was localized to the cell periphery and relatively concentrated around the growing ends of interphase cells and the mid-region of mitotic cells [7]. Wang *et al*. also reported that the GFP-fusion Rho3 protein was localized to the plasma membrane and the division site [8]. Our experiments were performed with the GFP-tagged Rho3 expressed chromosomally under the *nmt1* promoter. Notably, under conditions of *nmt1* promoter repression, GFP-Rho3 was localized in the dot-like structures observed in the cytoplasm (Figure 6, arrowheads) as well as at the plasma membrane and the division site (Figure 6, arrows). We examined whether the dot-like fluorescence of GFP-Rho3 co-localized with FM4-64 during an early stage of endocytosis. After 5 min of dye uptake, most of the GFP-Rho3 dot-like structures co-localized with FM4-64-positive structures (Figure 6, arrowheads; Merge). Thus, like Apm1, Rho3 localizes at Golgi/endosome structures in addition to the plasma membrane and the division site, which is consistent with its suggested additional role in Golgi/endosome trafficking. It should be noted that the GFP-Rho3 construct used by Wang *et al.*, supposedly utilizes a methionine distinct from that presented by the Sanger Centre (See Discussion). To allow colocalization with GFP-Rho3, we tagged Apm1 with the fluorescent epitope mCherry and the resultant Apm1-mCherry protein was detectable at the dot-like structures and the nucleus, which was the similar to the findings with the GFP-tagged version of Apm1 [9]. When GFP-Rho3 and Apm1-mCherry were co-expressed in the wild-type cells, some of the punctuate structures of GFP-Rho3 co-localized with the Apm1 dots in the cytoplasm (Figure 6B).

### Rho3 shows functional interaction with the AP-1 complex in *S. pombe*

**Figure 7:**
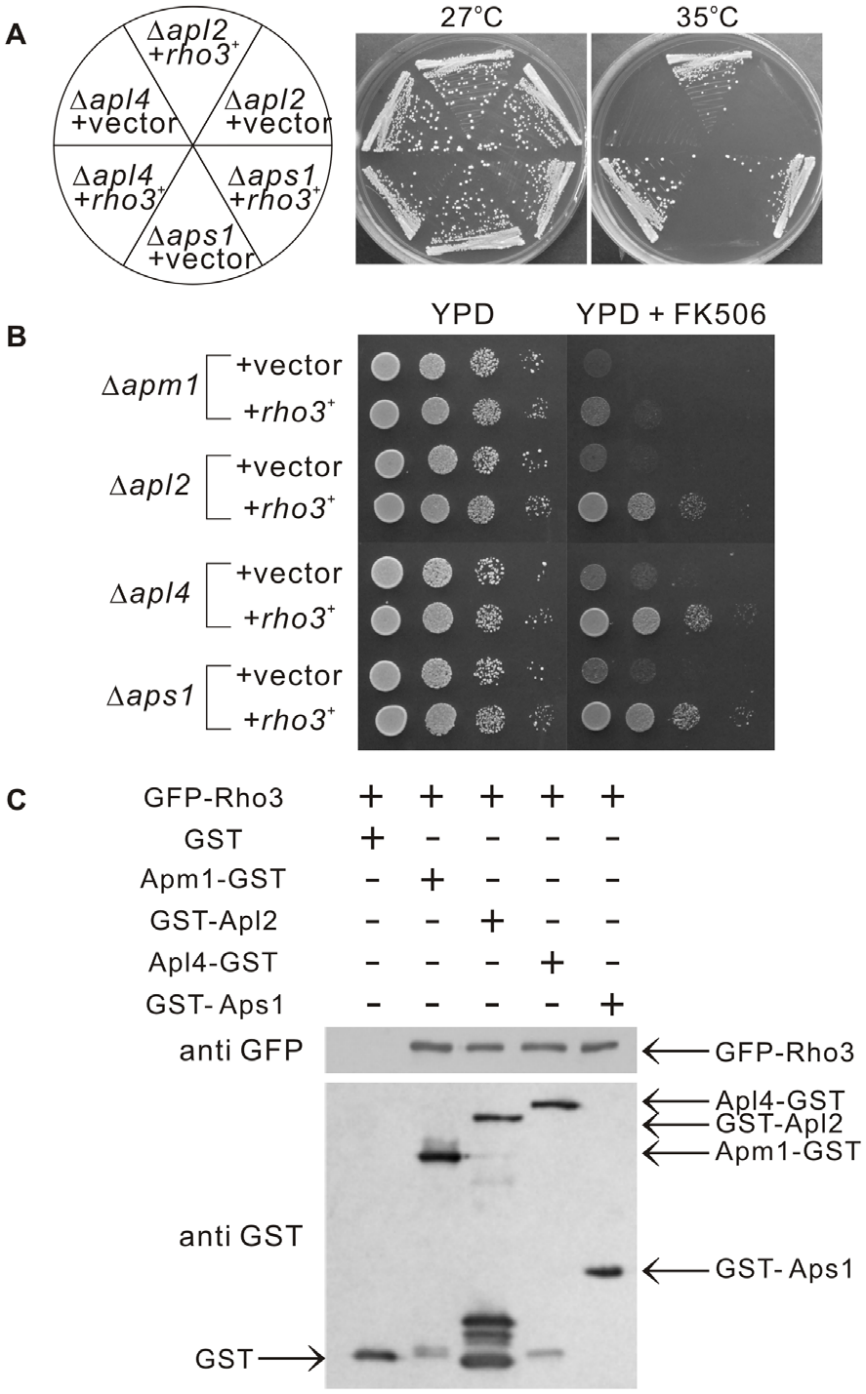
Functional interaction of Rho3 with the clathrin adaptor-protein complex. (A) Rho3 suppressed the temperature-sensitive growth of deletion mutants of individual adaptin subunit of the AP-1 complex. The Δ*apl2*, Δ*apl4*, and Δ*aps1* cells were transformed with either the pDB248 multicopy vector or with the *rho3^+^*-containing vector. The cells were then streaked onto plates containing YPD, and then incubated at 27°C for 4 d or at 35°C for 3 d, respectively. (B) Rho3 suppressed the immunosuppressant sensitivity of deletion mutants of individual adaptin subunit of the AP-1 complex. Cells as indicated in (A) were spotted onto YPD or YPD plus 0.5 µg/mL FK506 and incubated at 27°C for 4 d. (C) Binding assay involving Rho3 and the 4 subunits of the AP-1 complex. GST pull-down experiments performed with GST alone or Apm1-GST, GST-Apl2, Apl4-GST or GST-Aps1; cells expressing GFP-Rho3 were collected, and the lysates were incubated with purified full-length Apm1 fused GST, GST-Apl2, Apl4-GST or GST-Aps1 or the control GST protein. GST and GST-tagged adaptin subunits were precipitated by glutathione beads, washed extensively, subjected to SDS-PAGE, and immunoblotted using anti-GFP or anti-GST antibodies.

In a recent study, Ma *et al.*, reported that deletion mutants of AP-1 adaptin subunits display distinct phenotypes in *S. pombe* [23]. This prompted us to examine whether Rho3 overexpression can suppress the phenotypes of the deletion strains of other subunits of the AP-1 complex. For this purpose, the deletion strains of each individual subunit of the AP-1 complex, Apl2 (β), Apl4 (γ), and Aps1 (σ), were transformed with the plasmid containing the multi-copy *rho3* ^+^ gene. As shown in Figure 7A, Rho3 overexpression suppressed the temperature sensitivity of all the deletion strains harbouring the control vector. We also examined the effect of Rho3 overexpression on the immunosuppressant sensitivity of all the deletion strains of each individual subunit, and the results showed that the immunosuppressant sensitivity of all the deletion strains was suppressed by Rho3 overproduction (Figure 7B). It should be noted that in the cells grown on YPD plates, Rho3-mediated suppression in the Apm1 deletion cells was less prominent than that in the mutants for other subunits of the AP-1 complex.

Next, we examined if Rho3 can bind to each subunit of the AP-1 complex. To this end, we fused wild-type Rho3 to GFP and overexpressed the fusion protein by using an inducible *nmt1* promoter; the induced cells were used to prepare lysates. These lysates were then used in binding experiments where purified full-length Apm1, Apl2, Apl4, and Aps1 were fused to GST or the control GST protein. The results showed that the Rho3 protein bound to each of the subunits of the AP-1 complex (Figure 7C).

## Discussion

### Rho3 Plays a Role in Golgi/endosome trafficking

In the present study, we present several lines of evidence that suggests a novel role of Rho3 in Golgi/endosome trafficking on the basis of our discovery of the functional link between Rho3 and genes encoding adaptin. In our previous study, we established that the clathrin-adaptor complex is required for Golgi/endosome trafficking in *S. pombe* [9]. In this study, we showed that the overexpression of Rho3 suppressed the phenotypes of Δ*apm1* cells including defects in membrane trafficking and varied drug sensitivity. Furthermore, *rho3* deletion cells closely resemble *apm1* deletion cells in terms of the presence of multi-lamellar Golgi, accumulation of abnormal Berkeley bodies, and vacuole fragmentation, which was observed by electron microscopy and drug and Cl^−^ sensitivity. Results of the phenotypic analysis were supported by the co-localization of Rho3 with FM4-64 dots at the Golgi/endosomes as well as by the co-localization of Rho3 with Apm1 at the dot structures in the cytosol. Thus, Rho3 not only regulates secretion but it is also involved in the Golgi and vacuole function.

Intracellular localization of Rho3 in fission yeast has been reported by Nakano *et al*., and Wang *et al.* [7], [8]. Nakano *et al.* reported that Rho3 localized to the cell periphery by utilizing antibodies raised against Rho3 in budding yeast [27], and hence, there is an apparent difference between our methodology and the methodology used in their study [7]. In this study, we utilized GFP-fused Rho3 to visualize the Rho3 protein. Moreover, although immunofluorescent localization of Rho3 at sites other than the cell membrane and the division site was not reported, punctuate structures were detected throughout the cytosol [7]. However, it remains unclear whether these dots visualized by using budding yeast anti-Rho3 antibodies correspond to the Golgi/endosomes in *S. pombe*. Wang *et al.* reported that the GFP-Rho3 fusion protein localized to the plasma membrane and the division site and that no dot-like structures were observed in the cytosol [8]. The differences between the localization of GFP-Rho3 in the study reported by Wang *et al*. and our study might be attributable to the constructs used for the Rho3 ORF. We used the coding sequence of *rho3* ^+^ in the current Sanger Institutes database (http://old.genedb.org/genedb/Search?submit=Searchfor&name=rho3&organism=pombe&desc=yes&wildcard=yes) (see Materials and Methods). Although Wang *et al.* did not report any information about the primers used to construct the Rho3 ORF, the position of the first methionine used for the Rho3 ORF appears to be different from that of the methionine presented by Sanger Institute, which can be determined by the number of the reported amino acids that appeared in the paper; Rho3G25 corresponds to G22 in the database, and Rho3T30 corresponds to T27 in the database [8]. Furthermore, the primer sequence used by Wang *et al*. to disrupt the *rho3^+^* gene includes the flanking region just before the methionine (ATG) that localizes 148 bases upstream of the first methionine of the *rho3^+^* coding sequence in the current database [8]. The differences in the localization of the protein might be attributable to the addition of some amino acids to the N-terminus of Rho3, although it did not affect the function of Rho3 to complement the *rho3* null cells [8].

### Adaptin and Rho3-mediated membrane trafficking

How *rho3^+^* can suppress the phenotypes of not only the *apm1-1* mutant but also those of the *apm1*-delta cells remains unclear. The remarkable similarity of the phenotypes of the Δ*rho3* cells, including defects in membrane trafficking and drug sensitivity, with those of the Δ*apm1* cells, together with co-localization and binding of these proteins, give strong *in vivo* support for the notion that Apm1 serves as an effector of Rho3 in membrane trafficking at Golgi/endosomes. However, Rho3 overproduction suppressed the deletion phenotypes of all the subunits of the clathrin AP-1 complex, indicating that Rho3 exerts its effects in the absence of the AP-1 complex. In budding yeast, Rho3 was isolated as a suppressor of the cold-sensitive Rab GTPase mutant, *sec4-P48*, and 4 distinct components of the exocytic machinery, Sec4, Sec9, Sro7, and Sso2, can act as suppressors of rho3 deletion mutants [4]. In fission yeast, Rho3 was isolated as a multi-copy suppressor of *sec8-1*, a mutant allele of a component of the exocyst complex, and high dosage expression of Rho3p was unable to suppress the *sec8* null mutant cells, thus raising the possibility that Sec8 serves as a downstream target of Rho3 in secretion, although Wang *et al*. failed to detect a physical interaction between the exocyst proteins and Rho3 in immunoprecipitation experiments [8]. The exocyst complex is highly conserved from yeasts to mammals, and is involved in the late stages of exocytosis, by targeting and tethering post-Golgi vesicles to the plasma membrane [24]-[26]. Thus, the overexpression of Rho3 may stimulate secretion via the components of the exocyst (e.g., EXO70) by locally increasing the activity of the exocytic apparatus at sites occupied by GTP-bound Rho3 protein, which causes suppression of mutant strains in both yeasts that show exocytosis defects including the *apm1* deletion cells. However, it should be noted that overexpression of Rho3 only partially suppressed the secretion defects observed in the *apm1*-deletion cells. In contrast, Rho3 overexpression markedly suppressed the temperature-sensitive growth of the *apm1*-deletion cells, thereby suggesting that the mechanism of suppression by Rho3 also involves Rho3 function in addition to secretion.

We also investigated the genetic interaction between Rho3 and Apm1 by crossing Δ*apm1* and Δ*rho3* cells. However, we were unable to obtain double mutants. This finding indicates that Δ*apm1* and Δ*rho3* cells are synthetically lethal (data not shown), thereby suggesting that Rho3 regulates both secretion and Golgi/endosomes by influencing membrane trafficking in co-operation with Apm1. The synthetic lethality between *apm1*-delta and *rho3*-delta mutants can be explained by lowering of secretory machineries, including the AP-1 complex and exocyst complex, in the double mutant. However, overexpression of the exocyst genes, such as *sec8*, or the components of the AP-1 complex genes, failed to suppress the Apm1 deletion (data not shown), further supporting the hypothesis that suppression of *apm1* deletion might be achieved mainly through a mechanism other than stimulation of secretion.

These findings suggest that Rho3 may also exert its function partly through its target protein(s) involved in Golgi/endosome regulation. The physical interaction between Rho3 and Apm1 was analyzed by performing GST-pulldown experiments and affinity capillary electrophoresis assay. Although these experiments suggest a physical interaction between Apm1 and Rho3, our method could not exclude the possibility that the interaction is indirect. The formation of the AP-1 Rho3 complex can be achieved through a direct interaction between Rho3 and the target protein, and Rho3 exerts its Golgi/endosome function through its target protein(s) in the Golgi/endosomes. In higher eukaryotes, RhoB regulates endosome transport by promoting actin assembly on endosomal membranes through Dia1; RhoD regulates endosome dynamics through Diaphanous-related Formin and Src tyrosine kinase [3]. Interestingly, the Rho3 of fission yeast also binds to Formin and regulates polarized cell growth by controlling both the actin cytoskeleton and microtubules [7]. Thus, the Rho3 of fission yeast may play the same role as that played by RhoD in regulating Golgi/endosome organelle dynamics in higher eukaryotes. Consistent with this notion, Rho3 overexpression affects the abnormal accumulation of endosome structures in Δ*apm1* cells (Figure 2B). Future studies should unravel how Rho3 overexpression suppresses *apm1* mutant phenotypes associated with Golgi/endosomes by identifying the target protein(s) and unravelling the molecular nature of the interactions between Rho3 and its binding partners.
